# Environmental Competencies in Nurses and Undergraduate Nursing Students Related to the Effects of Climate Change on Older People’s Health

**DOI:** 10.3390/nursrep16050158

**Published:** 2026-05-08

**Authors:** Eva M. Montoro-Ramírez, Isabel M. López-Medina, Daniel Puente-Fernández, Laura Parra-Anguita

**Affiliations:** 1 Department of Nursing, Faculty of Health Sciences, Nursing and Innovation in Health Care Research Group (CuiDsalud CTS 464), University of Jaen, Las Lagunillas S/N, 23071 Jaen, Spain; emmontor@ujaen.es (E.M.M.-R.); lparra@ujaen.es (L.P.-A.); 2 Department of Nursing, University of Granada, 18071 Granada, Spain; danielpuentefdz@ugr.es; 3 Instituto de Investigación Biosanitaria ibs. Granada, 18012 Granada, Spain

**Keywords:** environmental competencies, clinical competence, knowledge, skills, attitude, climate change, older people, aged, nurses, nursing students

## Abstract

**Introduction:** Climate change is increasingly affecting the health of older people. This study aimed to determine the knowledge, skills, and attitudes of nurses and undergraduate nursing students regarding the effects of climate change on older people’s health. **Material and Methods:** A descriptive cross-sectional study was conducted between January and April 2024 with 708 participants (210 nurses and 498 undergraduate nursing students). The Nursing Competencies Questionnaire on Environmental Health of Older People (NCQ-OPEH) was used to assess environmental competencies. Descriptive values were calculated and interrelationships between knowledge, attitudes, and skills were analysed. **Results:** A total of 115 nurses (54.75%) and 185 students (37.15%) demonstrated good-excellent knowledge. Similarly, a higher percentage of nurses (50.77%) reported better perceived skills than students (42.52%). However, the majority of both samples (98.97% and 87.85%, respectively) had good to excellent attitudes. These differences were significant for knowledge (*p* < 0.001) and attitudes (*p* = 0.013) but not for skills (*p* = 0.054). Furthermore, a significant relationship was found between prior education on climate change and health and greater knowledge (*p* = 0.019) and skills (*p* = 0.027) among nurses and better skills and attitudes (*p* < 0.001 in both) among nursing students. **Conclusions:** Nurses have better environmental competencies than undergraduate nursing students. Therefore, it is important to include education on climate change and older people’s health to be included in the academic curriculum of university nursing degrees. Nurses also need to reinforce these competencies through specific educational programmes, ensuring that clinical practice effectively adopts an environmental health approach to the care of older people.

## 1. Introduction

Nowadays, there is growing concern about understanding how climate change and global warming will affect the ageing population [1]. The physiological changes associated with the ageing process increase the vulnerability to harmful stimuli, especially in cases of comorbidity, chronicity, and/or polypharmacy. As a result, this collective is particularly vulnerable to the effects of climate change [2]. There is also a relationship between climate change and the mental health of the older population, potentially inducing new or aggravating existing mental health conditions or aggravating existing ones [3].

The Sustainable Development Goals (SDGs) represent a global action plan to address the challenges that climate change presents for the health of older people. Nurses contribute to improving the health and well-being of older people at risk from climate change (SDG 3) and enable climate action (SDG 13) through sustainable, quality nursing care that strengthens resilience and adaptability to the dangers associated with climate change [4]. In line with the SDGs, the concept of planetary health highlights the close relationship between human health and the ecological systems that support life. It thus emphasises the need for healthcare professionals to address the profound impacts that environmental changes have on health. For this reason, nurses need to develop specific environmental competencies—encompassing knowledge, skills and attitudes—facilitated through Education for Sustainable Development (ESD) using the Planetary Health Model as a framework [5,6]. The assessment of environmental competencies in this study is based on a holistic definition of professional competence, which goes beyond the mere acquisition of information. According to Guzmán et al., competence is conceptualised as the integrated mobilisation of knowledge (cognitive), skills (psychomotor) and attitudes (affective) to address complex real-world challenges. In the context of nursing and global health, this triad ensures that professionals not only understand the connection between climate and health but also have the clinical skills and ethical commitment (attitude) necessary to implement sustainable interventions [7].

This educational approach empowers nurses to act as agents of change, ensuring the provision of sustainable healthcare whilst protecting vulnerable populations from the risks posed by climate change. Nursing professionals must be able to establish adaptation, mitigation, and resilience strategies to this end. It should also be taken into account that the healthcare sector contributes to climate change through its consumption of energy and resources and the generation of waste. In fact, its climate footprint accounts for 4.4% of global net emissions [5]. Healthcare professionals, particularly nurses, are on the front line when it comes to facing the health impacts of climate change on older people, whether direct (disasters such as floods) or indirect (increased demand for emergency services and hospitalisations). As climate change accelerates, the need to treat climate-related illnesses in this population group will also increase [6]. Nurses hold a position of trust in the community and can therefore play an influential role by providing information on the health effects of climate change. They can help communities through education on prevention and adaptation to impacts, as well as understanding the potential risks [8]. However, turning strategies into concrete actions requires going beyond mere theoretical knowledge. Through the use of the KSA (knowledge, skills, and attitudes) framework, we can deconstruct nursing competence into its fundamental components: a solid understanding of climate-related risks (knowledge), the practical ability to intervene during heatwaves or environmental crises (skills) and a professional commitment to sustainable healthcare (attitudes). Identifying exactly where these gaps lie is the only way to design the type of education programmes that will keep older people safe and well cared for in an increasingly unpredictable climate [9]. The implementation of the KSA framework is fundamental to both nursing education and professional practice, providing a robust, multidimensional structure for achieving holistic clinical competence. For nursing students, this model serves as a vital pedagogical roadmap that transcends rote academic learning, synthesising clinical reasoning with technical proficiency and ethical conduct from the very outset of their training. For nurses, the KSA framework facilitates critical self-appraisal and continuous professional development; in an increasingly volatile healthcare landscape—defined by climate-related health crises and rapid technological shifts—the constant refinement of theoretical knowledge must be seamlessly integrated with advanced practical expertise and a resilient professional attitude [10,11].

While the role of nursing in addressing climate-sensitive pathologies has gained increasing recognition, the current body of literature exhibits critical lacunae. Primarily, the systematic assessment of nursing-specific competencies—comprising the knowledge, attitudes, and skills fundamental to environmental health—as well as professional preparedness, remains critically under-examined [12,13,14]. Notably, there is a profound lack of empirical evidence concerning the specific implications for the geriatric population. Furthermore, to the best of our knowledge, no comparative analyses currently exist evaluating these competencies between practising registered nurses and nursing students. Finally, this evidentiary gap is particularly salient within the context of higher education, where the integration of climate change and environmental health into nursing curricula persists as a significant challenge highlighted across recent scholarship [15,16].

Therefore, it is essential that university students receive ESD through the incorporation of skills to promote planetary health. It is necessary to provide and instruct nurses and nursing students in the knowledge, skills, and attitudes required to develop actions aimed at finding solutions to climate change [7,8,17]. In short, it is up to the nursing profession to make significant changes to improve society’s response to climate change and drive the transformation needed to achieve a healthy future [18].

Although there is growing evidence of the interconnection between climate change and the health of older people, a considerable gap exists in the knowledge, skills, and attitudes of nurses and nursing students to address these issues. Many healthcare professionals have no formal training in climate-related health and care provision; therefore, they are unprepared and only perform half of the tasks involved in environmental measures [19]. Few nurses understand why extreme weather events are linked to climate change and how they affect the health of older people. Consequently, they are unaware of strategies or interventions to reduce the impact on this population [20]. Furthermore, there are currently no clear guidelines for the development of curricula that integrate climate change into university nursing education, despite the fact that the SDGs clearly highlight the need for education for sustainable development (ESD) that aims to maintain planetary health [21]. However, nursing students are generally not adequately trained in the impacts of climate change on health and the appropriate response of the nursing profession [8]. Therefore, this study aimed to determine the knowledge, skills, and attitudes of nurses and undergraduate nursing students regarding the effects of climate change on the health of older people. In addition, this study aims to determine these competencies based on various sociodemographic variables. The hypothesis was established that nurses obtain significantly higher levels of environmental competencies (measured as knowledge, skills, and attitudes) than nursing undergraduates for the care of older people exposed to climate-related risks.

## 2. Materials and Methods

### 2.1. Study Design

This research is a descriptive cross-sectional study. The STROBE (Strengthening the Reporting of Observational Studies in Epidemiology) statement for cross-sectional studies was used to prepare the report [22] (Supplementary Materials S1).

### 2.2. Participants

To estimate the required sample size in Survey Research, Cochran’s formula was used, taking into account a 95% confidence level (Z = 1.96), a 5% margin of error (E = 0.05) and assuming an expected variability of 50% (*p* = 0.5). Therefore, as the size of the target population was unknown, a sample of 385 participants was obtained [23]. The inclusion criteria for nurses were that they were currently working in primary care, nursing homes or hospitals, and were based anywhere in the country. For nursing students, they had to be in any of the four years of the nursing degree programme and be enrolled at one of the participating universities.

For this study, a non-probabilistic convenience sample of 708 participants (210 nurses and 498 undergraduate nursing students) was chosen, as this approach enabled valuable information to be obtained from participants who met the inclusion criteria and were highly willing to take part.

### 2.3. Data Collection

Various strategies were employed for data collection to ensure that the sample was as representative as possible between January and April 2024. To recruit nurses, the questionnaire was shared via social media platforms, including Twitter, Instagram and WhatsApp, whilst emails were also sent from hospitals to nursing staff. Undergraduate nursing students were selected from three different Spanish universities during their clinical placements to ensure maximum participation. Furthermore, representation from all four years of the Bachelor’s degree in Nursing was ensured. The link to the questionnaire hosted on the SurveyMonkey^®^ online platform was provided in both samples.

### 2.4. Research Instrument

Participants completed the NCQ-OPEH (Nursing Competence Questionnaire on the Environmental Health of Older People), a tool designed to assess the knowledge, skills and attitudes of both nurses and nursing undergraduates regarding the effects of climate change on the health of older people. The instrument consists of a knowledge questionnaire, the KQ-OPEH (11 items with ‘true/false/don’t know’ response options), a skills scale, SS-OPEH (13 items with response options on a 1–5 Likert scale ranging from totally disagree to totally agree), and an attitude scale, AS-OPEH (11 items with the same response options on a Likert scale). During its development, a panel of 13 experts was consulted, establishing an Aiken’s V value of 0.80 as the consensus. A pilot test was also conducted to ensure the items were interpreted correctly. Lastly, this tool was psychometrically validated using Item Response Theory; specifically, the Rasch Model was used for the knowledge questionnaire and the Andrich Scale Model for the skills and attitudes scales. It demonstrates strong internal consistency across each of its components (Cronbach’s α values of 0.68, 0.91 and 0.93, respectively). In both models, all items showed adequate fit statistics, with infit and outfit mean values of 1 (0.57 to 1.58). Furthermore, the item- and person-discrimination index values were sufficiently high (above 2) to ensure the instrument’s potential to categorise different levels of environmental competence among nurses and nursing students.

The categorisation of scores was intentionally rigorous, reflecting the high stakes of clinical practice when safeguarding vulnerable older populations against environmental health threats. For the knowledge component, a five-tier classification was adopted; scores representing ‘excellent’ (>90%) or ‘very good’ (80–89%) mastery were defined as the requisite standard for safe, independent decision-making. Conversely, any score falling below 60% was classified as insufficient, as significant theoretical gaps in climate-related pathophysiology could compromise patient outcomes. A lower score (59–40%) indicates insufficient knowledge, as it is assumed that there is a deliberate and coherent understanding of the subject matter; furthermore, a score below 40% would suggest significant gaps in the identification of risks, given that the relationship between climate variables and the health of older people is multifaceted and complex. For both skills and attitudes, the same five categories were established, but on these scales, the competency threshold was set at a minimum of 70% to be considered “good”, as this standard is essential to ensure that the training received does not remain on an abstract level, but is translated into effective, empathetic and technically sound care for this vulnerable population [10,24].

These described cutoff points are based on previous studies that use the KSA framework in nursing and climate change, employing very similar hierarchical categorisations [14,25,26]. By applying this hierarchical classification, the study can clearly distinguish between a superficial awareness of climate issues and the robust professional competence required by current global health standards.

### 2.5. Data Analysis

Sociodemographic variables were also collected from participants, such as age, gender, previous education on climate change and health, workplace, course taken, and completion of healthcare internships, to study whether there are differences in competencies based on these variables. In addition, frequencies and percentages were calculated for the qualitative variables, and measures of central tendency (mean and standard deviation) for the quantitative variables.

To compare the difference in scores between nurses and students and between those who had previous education on climate change and those who did not, the non-parametric Mann–Whitney U statistic was used, since the Kolmogorov–Smirnov test indicated that the data deviated from a normal distribution. The effect size was calculated using the biserial rank correlation. To assess whether there were significant differences between the mean scores for different sociodemographic variables (gender, workplace, and course enrolled), the Kruskal–Wallis test was used as the data did not meet the assumption of homogeneity of variances through Levene’s test. The Rank ε^2^ value was calculated to indicate the effect size. In cases where a statistically significant relationship was found, Dunn’s post hoc test was performed by applying the Bonferroni adjustment to the *p*-values. The analysis was completed by examining Spearman’s rho to assess the correlation between knowledge, skills, and attitudes, as well as age, in each population group.

The free software JASP (Version 0.95.4) was used for both descriptive statistics and hypothesis testing.

## 3. Results

### 3.1. Demographic Data

Among the nurses’ sample, respondents were from primary care, nursing homes, and hospital care. Undergraduate nursing students from the four years of the Nursing Degree programme were also surveyed. The majority of the total sample were women (80.93%) and had no previous education on climate change and health (66.81%), with the latter percentage being much higher in nurses (Table 1).

### 3.2. Knowledge on Climate Change and the Health of Older People

A total of 708 participants completed the knowledge questionnaire. With regard to knowledge, less than half of the complete sample (42.37%) demonstrated good to excellent knowledge: 54.75% of nurses and 37.15% of undergraduate nursing students (Table 2).

The item with the highest number of correct answers was K2 *(*‘*Socioeconomic factors affect older people’s ability to adapt to climate change risk’*), with 92.38% of nurses and 88.55% of nursing students. In contrast, item K5 (‘*Extreme heat is the climate change effect that most affects older people’s health’*) was the most mistaken, with 87.62% nurses, and 67.07% students choosing the wrong option. Finally, the least known item was K3 *(‘Climate change consequences affect older women’s health more than that of same-age men’*), with 53.33% nurses and 60.44% students.

The mean score obtained in the knowledge questionnaire was 6.04 (SD = 1.93) out of a maximum total of 11, with 6.58 (SD = 1.79) for nurses and 5.82 (SD = 1.95) for nursing students. The difference of 0.73 points more on average in the knowledge questionnaire for nurses than for university students was significant, although with a small–moderate effect size 0.23 (SE = 0.05) (Table 3).

Nurses with prior training on climate change and health had greater knowledge than those who did not (0.88 points higher on average), with a small–moderate effect size of 0.28 (SE = 0.14). In contrast, no significant differences were found between university students based on whether or not they had received such training (Table 3).

Although differences in knowledge were found based on gender, these differences were not significant in either nurses or students. This was not the case with regard to age, as a weak correlation with knowledge was observed in both populations, but in the opposite direction. Among nurses, the older they were, the less knowledge they had, whereas among university students, the older they were, the greater their knowledge. (Table 3).

Knowledge of nurses also varied slightly depending on the workplace, ε^2^ = 0.05 (95%CI, 0.003–0.113) (Table 3). Dunn’s post hoc comparisons showed that nurses working in other types of care or education centres scored 2.76 points higher on the knowledge questionnaire than those working in primary care and 2.07 points higher than hospital nurses. Similarly, nurses at nursing homes obtained an average of 2.43 points more than those in primary care and 1.97 points more than those working in hospitals. However, after applying the Bonferroni correction, only the first difference in the knowledge questionnaire score was significant.

Finally, among undergraduate nursing students, a slightly significant correlation was found between having completed assistance practice in a health care centre or hospital (effect size 0.19; SE = 0.06) and being in a more advanced academic year with a higher knowledge about climate change and the health of older people, ε^2^ = 0.02 (95%CI, 0.006–0.061) (Table 3). In general, the higher the course level, the more the knowledge, although only the difference between the first and third courses was significant in Dunn’s post hoc analysis (3.24 points more).

### 3.3. Skills on Climate Change and the Health of Older People

A total of 672 participants (197 nurses and 475 students) completed the skills scale. Slightly less than half of the sample (44.94%) perceived that they had good to excellent skills in caring for older people at climate risk. The group of nurses reported 50.77% good to excellent perceived skills, whereas the nursing students reported 42.52% (Table 4).

For both populations, the item with the highest perceived skill was S7 (‘*I am able to assess the housing conditions that pose a risk to older people in case of extreme temperatures’*) (3.80, SD = 0.87), while the item with the lowest perceived skill was S5 (*‘I am able to identify the climate change effects that may affect the mental health of older people’*) (2.90, SD = 1.08).

The mean score for the skills scale was 44.25 (SD = 8.73) on a maximum of 65 points, with the mean for nurses being 44.86 (SD = 7.97) and students being 43.99 (SD = 9.02). However, this difference in mean scores between the two populations was not statistically significant according to the Mann–Whitney U test (Table 5).

Prior education on climate change and health resulted in nurses and nursing students reporting greater perceived skills in caring for older people at climate risk, with higher mean scores of 4.08 and 3.65 on the SS-OPEH, respectively (Table 5). In both cases, the effect size was small to moderate, at 0.27 (SE = 0.14) and 0.26 (SE = 0.05), respectively.

As with knowledge, the statistics revealed no significant differences between gender-based skills in either population. Being older and possessing better skills are not related among nurses, whereas older university students have slightly greater skills (Table 5).

The Kruskal–Wallis test showed statistical differences in nurses’ skills based on their workplace (Table 5), with a moderate effect size (ε^2^ = 0.07 (95%CI, 0.019–0.172). In particular, Primary Care nurses have higher perceived skills than hospital care nurses, with an average of 1.98 points more on AS-OPEH. Primary care nurses obtained higher scores than the rest, although the Bonferroni corrections resulted in non-significant values.

In addition, nursing students reported a moderate correlation between completed assistance practice in health centres or hospitals (effect size 0.39; SE = 0.06) and academic year with perceived environmental skills (ε^2^ = 0.08 (95% CI, 0.051–0.139) (Table 5). Those students who have completed internships have more perceived skills (5.31 higher score). Third-year students report higher perceived skills than first-year students (an additional average of 5.86 points) and second-year students (an additional average of 4.46 points).

### 3.4. Attitudes on Climate Change and the Health of Older People

In total, 648 participants (195 nurses and 453 students) completed the attitude scale. The vast majority of participants (91.18%) had a good to excellent attitude towards the effects of climate change on the health of older people. Among nurses, the proportion in these categories was 98.97%, whereas among students, it was 87.85% (Table 6).

In both populations, the participants showed a more positive attitude towards item A6 (‘*It is important that health professionals are aware of the consequences of the effects of climate change on the health of older people’*) (4.41, SD = 0.71), while the greatest disagreement was shown towards item A8 (‘*Health professionals have the responsibility to address climate change impacts on the health of the older population*’) (4.06, SD = 0.82).

The attitude scale obtained an average score of 46.81 (SD = 6.41) out of a maximum total of 55 points, with significantly higher scores for nurses (47.02, SD = 4.99) than for nursing students (46.33, SD = 6.89) (Table 7). The statistical significance was weak with an effect size of 0.11 (SE = 0.05).

Only a weak to moderate significant association was observed between prior education on climate change and health and better attitudes among nursing students, 2.62 points higher in AS-OPEH with an effect size of 0.20; SE = 0.06 (Table 7).

On this occasion, no relationship was found between gender and age and having better attitudes towards caring for older people at risk from climate change in either of the two populations, as the differences were not significant (Table 7).

Similarly, no significant relationship was reported between the attitude level and the nurses’ workplace (Table 7).

On the other hand, among university nursing students, a slight relationship was found between having external practices (effect size 0.16; SE = 0.06) and the academic year completed (ε^2^ = 0.02 (95%CI, 0.005–0.068) with better environmental attitudes (Table 7). More specifically, 1.73 higher score than those who have not practised yet. Attitudes also increase as the year of study progresses, with a significant difference between the third and first years in terms of the AS-OPEH score (2.76 points higher on average).

### 3.5. Overall Environmental Competencies

Statistically, knowledge and skills are positively correlated among nurses (weak correlation). In other words, the more knowledge they have about the effects of climate change on the health of older people, the greater their perceived skills in caring for them. However, no significant relationship was found between knowledge and attitudes or between skills and attitudes (Table 8).

Knowledge, skills, and attitudes are positively correlated in undergraduate nursing students (weak association): the greater the knowledge, the more skills and better the attitudes the students have. Furthermore, the more perceived skills they have, the better their attitudes are towards the role of nursing in the effects of climate change and the environmental health of older people (Table 8).

## 4. Discussion

The results of our study partially support the main hypothesis, since although nurses have greater environmental competencies in older people’s environmental health than undergraduate nursing students, this is statistically significant in terms of knowledge and attitudes, but not for skills. In general, nurses have moderate knowledge and perceived skills, whereas students have insufficient or poor skills. In contrast, both groups have very high attitudes towards the effect of climate change on the health of older people. These findings highlight that those competencies in older people’s environmental health represent a new area of nursing practice; consequently, although attitudes towards this new approach to healthcare are becoming increasingly positive, there remains a need to develop the necessary knowledge and skills, particularly among students. The minimal difference in skill levels between nurses and students may be due to the fact that these skills are relatively new to nursing practice in the study context; consequently, being an experienced nurse does not necessarily guarantee greater mastery of these skills. However, a larger sample size of nurses could confirm whether the greater skills demonstrated by this group compared to the student group are statistically significant.

No previous research has analysed nurses’ environmental competencies regarding the health of older people [9]. Nevertheless, most studies on nurses suggest that their knowledge about climate change and health is moderate, as shown in our research [27,28]. Other researchers, however, found that health professionals had insufficient knowledge about the scientific aspects of climate change [14,29,30] or more specific aspects of it [31,32]. A study showed that although nurses had good knowledge about climate change and health, more than half were unable to recognise the older population as a group that is particularly vulnerable to its effects [33]. Regarding undergraduate nursing students, our report indicated less knowledge than nurses about the environmental health of older people and the climate risks to which they are exposed. This is consistent with studies that have measured knowledge about climate change and health among nursing students, finding high percentages of insufficient knowledge [34,35,36,37,38]. This lack of knowledge may be due to the fact that the most common sources of information on the consequences of climate change were not official, as various studies point out, nor were they through social media [9,29,34]. We only found greater knowledge in higher-level courses at universities where they had already received some training on the subject, as Aronsson reported [39]. This finding is consistent with the educational system in the study context, given that students receive training on environmental health for older people in the third year of the nursing degree. We therefore believe it is important to gradually incorporate environmental competencies into nursing degree programmes, both in theoretical training and in clinical simulations and practical training in healthcare settings. As in our research, some studies found a positive correlation between students’ knowledge and attitudes. This means that greater knowledge leads to better attitudes towards climate change and sustainable practices [37]. Some research has found a relationship between female gender and knowledge [35,36,40], although this was not the finding in our study, as in the case of researcher Çetin [38]. As in our results, studies have shown that prior education on climate change and health is associated with greater knowledge and attitudes [38]. One possible explanation could be that having studied the subject makes them more aware of the importance of the effects of climate change on health and, as a result, leads them to adopt more positive attitudes towards environmental health.

Our research showed that skills for caring for older people affected by climate change were greater among nurses, especially those working in primary care. However, although this difference was not statistically significant, it could suggest that primary care nurses might adopt a more holistic view of health, taking environmental factors into account. Although we have not found any articles on this vulnerable population, there is research that reveals the limited skills of nurses [14,41] and nursing students in relation to environmental health in general [34]. A positive correlation between knowledge and skills has been demonstrated [14]. This could explain the lack of skills due to a deficit of knowledge about climate change and its effects on health [38], which once again highlights the importance of specific training on older people’s environmental health, including training provided by healthcare institutions themselves. As with knowledge, this study found no significant differences in skills in terms of gender, either among nurses or nursing students. Although Álvarez-García [34] and Levine [42] found a higher perceived level of competence among male students, our results are consistent with a more egalitarian trend in nursing education. This discrepancy could be attributed to the standardisation of clinical training protocols, which ensures that the acquisition of competencies is independent of gender. Furthermore, the absence of a confidence gap in our sample may reflect a more objective self-assessment tool that minimises traditional gender-based biases in self-reported competence.

The two populations studied in this research demonstrated that the vast majority had excellent attitudes towards how climate change affects the health of older people. This result is repeated in practically all the studies found on environmental attitudes [14,30,33,34,43,44,45,46,47]. Although both demonstrated excellent attitudes towards climate change and the important role of nursing in preventing and mitigating its effects [39], most disagreed with the responsibility of healthcare professionals to address these effects through sustainable care. We found discrepancies between studies in which most nurses recognised that health systems contribute to climate change [33,44] and others in which only a minority were aware of the contribution of the health sector [32,41,48,49]. Although motivated, the participants admitted that they needed more knowledge and skills to respond appropriately [44,48]. Furthermore, several studies have suggested that, in general, there is a disconnect between nurses’ awareness of climate change and the implementation of sustainable practices. The main reason reported is the heavy workload they typically face on a daily basis [40,44,50]. The significance of the findings highlights the urgent need to reformulate current health policies, which often operate under a traditional biomedical model that ignores the patient’s environment. For clinical practice to effectively adopt an environmental health approach to the care of older people, it is imperative that government bodies develop guidelines that recognise environmental exposure as a modifiable risk factor. Policies are needed to fund the training of healthcare professionals in this area and to standardise environmental care processes for older people. Others emphasised meeting patients’ immediate needs rather than carrying out environmentally responsible practices [49,51]. Although both nurses and nursing students agreed on the need for healthcare professionals to be aware of the effects of climate change on the health of older people, the majority of participants in both groups in this study had not previously participated in training courses on climate change and health, with this figure being much higher among nurses, as reported by Abdelaziz et al. [14]. This reveals a significant challenge, as nurses require ongoing education on climate change, its impact on health, and the role of nursing in addressing the issue [41].

## 5. Limitations

The authors identified some aspects that could limit the results to a certain extent. Firstly, as this is a cross-sectional study, it is not possible to establish causal relationships, but only associations. Although associations between the variables have been identified, longitudinal studies are needed to determine how clinical practices in the field of environmental health evolve over time. Second, despite the significant findings, this study has limitations in terms of its external validity due to the use of non-probabilistic convenience sampling, particularly in the case of nursing students. This methodology may have introduced selection bias, attracting individuals with above-average literacy levels or interest in environmental issues. Consequently, any generalisation of these results should be made with caution. An attempt was made to minimise this by using a large sample of participants from different locations throughout the country to achieve the greatest possible representativeness. Furthermore, the results may have limited generalisability due to the specific geographical and cultural context of the sample. Regional health policies and nursing curricula vary considerably, which may influence how environmental health is integrated into clinical practice compared with other international settings. Moreover, voluntary participation in the study may have introduced selection bias (self-selection), such that people who already had a prior interest in environmental health or those who consider themselves more competent may have been more willing to participate, which could have led to an overestimation of the attitudes and skills reported. Similarly, as this study is based on self-reported data, it may be subject to social desirability bias; participants may have overestimated their clinical skills or expressed more favourable attitudes towards environmental health than those observed in real clinical settings. This bias was reduced by the participants’ complete anonymity (which also controls for researcher bias).

### Future Research

The approach for future projects focuses on creating materials and implementing educational interventions to increase the environmental competencies of nurses and nursing students for the care of older people at climate risk, as the results of this study and the literature suggest that basic nursing training or professional development does not adequately address climate change [27]. Training nursing professionals in climate-related issues is a key step towards modernising the profession. By including this focus in university curricula and research, clinical practice and health policies can be ensured to be aligned with the current planetary reality [52]. We found several actions undertaken in this regard in the literature, including strengthening the content of university degree programmes [53,54], creating online materials and modules for professionals [55], developing educational video games [56], simulation [57], and augmented reality [47].

Consequently, the present study may provide a roadmap for adaptive instructional design, ensuring that the educational workload is distributed and structured according to the specific weaknesses identified in each demographic profile. It provides the basis for identifying significant disparities in levels of knowledge, skills and attitudes concerning the care of older people at climate risk. For example, the presence of significant differences depending on the academic year or work environment provides a fundamental needs diagnosis for the design of training activities. The findings provide a precise tool for strategically tailoring future educational interventions: whilst knowledge gaps suggest a need for updated theoretical content, deficiencies identified in skill domains call for practice-based learning methodologies and contextualised clinical simulation.

Once nurses have acquired the necessary knowledge, their ability to apply it in clinical practice does not depend exclusively on having a positive attitude, as other external factors that go beyond personal motivation also play a role. Further research is needed to determine which strategies should be adopted in different healthcare settings to facilitate the implementation of environmental nursing skills.

## 6. Conclusions

Nurses have better environmental competencies than undergraduate nursing students, mainly in knowledge and skills. Prior education on climate change and health increases environmental competencies, such that greater knowledge leads to greater skills and better attitudes towards its effect on the health of older people. On the other hand, attitudes were excellent in both groups, reflecting the fact that they are aware of the vulnerability of this population and the need for health professionals to actively address this issue. There is a need to enhance nursing knowledge regarding environmental and health-related competencies in order to improve skills in caring for older people as a climate-vulnerable group. This training should be incorporated across the board into nursing degree programmes and should be sustained through specific training programmes throughout a nurse’s career. Our findings allow us to state that a positive attitude alone is not enough; we need well-trained professionals and coherent health policies to provide older people with healthcare based on prevention and specific care that addresses the effects of climate change on their health. Future research should focus on developing appropriate education programmes to respond to this need.

## Figures and Tables

**Table 1:** Participants’ sociodemographic characteristics.

Characteristic	Nurses	Nursing Students
n (%)	210 (29.66)	498 (70.34)
Mean age, years (SD)	43.81 (10.90)	22.94 (7.02)
Gender, n (%):		
*Male* *Female*	38 (18.10) 170 (80.95)	90 (18.07) 403 (80.92)
*Others*	2 (0.94)	5 (0.80)
Education in climate change and health, n (%): *No* *Yes*	191 (90.52) 19 (9.05)	279 (56.02) 219 (43.98)
Nurses’ workplace, n (%): *Primary Care* *Nursing Homes* *Hospital Care* *Other*	54 (25.71) 8 (3.81) 115 (54.76) 33 (15.71)	NA NA NA NA
Highest course enrolled, n (%): *First* *Second* *Third* *Fourth*	NA NA NA NA	162 (32.53) 184 (36.95) 133 (26.71) 19 (3.82)
Assistance practices, n (%): *No* *Yes*	NA NA	338 (67.87) 160 (32.13)

SD: Standard deviation; NA: not applicable.

**Table 2:** Level of knowledge of the participants.

Knowledge	Total Sample		Nurses		Students	
	n	%	n	%	n	%
Excellent (11 pts)	1	0.14	1	0.47	0	0
Very Good (9–10 pts)	75	10.59	28	13.33	47	9.44
Good (7–8 pts)	224	31.64	86	40.95	138	27.71
Not Enough (4–6 pts)	344	48.59	86	40.95	258	51.80
Poor (0–3 pts)	64	9.04	9	4.28	55	11.04

**Table 3:** Statistical tests for the KQ-OPEH.

	Nurses	Students
NURSES-STUDENTS Mann–Whitney U test	64,084 **	
PRIOR EDUCATION Mann–Whitney U test	2330 *	31,063
GENDER Mann–Whitney U test	3424.50	19,861
AGE Spearman rho	−0.17 *	0.13 *
WORKPLACE Kruskal–Wallis	11.05 *	NA
ASSISTANCE PRACTICES Mann–Whitney U test	NA	31,630 **
COURSE ENROLLED Kruskal–Wallis	NA	11.12 *

In bold, significant *p*-values; * *p* < 0.05; ** *p* < 0.001; NA: not applicable.

**Table 4:** Level of skills of the participants.

Skills	Total Sample		Nurses		Students	
	n	%	n	%	n	%
Excellent (59–65 pts)	33	4.91	6	3.05	27	5.69
Very Good (52–58 pts)	75	11.16	26	13.20	51	10.74
Good (46–51 pts)	194	28.87	68	34.52	124	26.11
Not Enough (33–45 pts)	310	46.13	81	41.12	229	48.21
Poor (13–32 pts)	60	8.93	16	8.12	44	9.26

**Table 5:** Statistical tests for the SS-OPEH.

	Nurses	Students
NURSES-STUDENTS Mann–Whitney U test	50,477	
PRIOR EDUCATION Mann–Whitney U test	2147.50 *	34,461 **
GENDER Mann–Whitney U test	2950	17,562
AGE Spearman rho	0.05	0.16 **
WORKPLACE Kruskal–Wallis	12.65 *	NA
ASSISTANCE PRACTICES Mann–Whitney U test	NA	33,450 **
COURSE ENROLLED Kruskal–Wallis	NA	38.15 **

In bold, significant *p*-values; * *p* < 0.05; ** *p* > 0.001; NA: not applicable.

**Table 6:** Level of participants’ attitudes.

Attitude	Total Sample		Nurses		Students	
	N	%	N	%	N	%
Excellent (50–55 pts)	240	37.03	76	38.97	164	36.20
Very Good (44–49 pts)	252	38.88	89	45.64	164	36.20
Good (39–43 pts)	99	15.27	28	14.36	70	15.45
Not Enough (28–38 pts)	52	8.02	1	0.51	51	11.26
Poor (11–27 pts)	5	0.77	1	0.51	4	0.88

**Table 7:** Statistical tests for the AS-OPEH.

	Nurses	Students
NURSES-STUDENTS Mann–Whitney U test	48,999 *	
PRIOR EDUCATION Mann–Whitney U test	1763.50	30,014.50 **
GENDER Mann–Whitney U test	2009.50	16,337.50
AGE Spearman rho	0.05	0.03
WORKPLACE Kruskal–Wallis	13.50	NA
ASSISTANCE PRACTICES Mann–Whitney U test	NA	25,763 *
COURSE ENROLLED Kruskal–Wallis	NA	8.80 *

In bold, significant *p*-values; * *p* < 0.05; ** *p* > 0.001; NA: not applicable.

**Table 8:** Knowledge-Skills-Attitude correlation.

	Spearman rho (*p* Value)	
	Nurses	Students
Knowledge-Skills	0.23 *	0.35 **
Knowledge-Attitude	0.13	0.12 *
Skills-Attitude	0.13	0.18 **

In bold, significant *p*-values; * *p* < 0.05; ** *p* > 0.001; NA: not applicable.

## Data Availability

The original data presented in the study are openly available in RUJA at https://hdl.handle.net/10953/6471. The instrument, the Nursing Competencies Questionnaire on Older People’s Environmental Health (NCQ-OPEH), can be consulted at https://hdl.handle.net/10953/7389.

## Supplementary Materials

The following supporting information can be downloaded at: https://www.mdpi.com/article/10.3390/nursrep16050158/s1, S1: STROBE Statement—Checklist of items that should be included in reports of cross-sectional studies.

## Public Involvement Statement

Nurses and nursing students were involved in this research through responding to the survey. This study was carried out in accordance with the principles outlined in the Declaration of Helsinki. The research was approved by the Andalusian Biomedical Research Ethics Committee (PEIBA) (code: Tesis-CCECCSM-2019/0902-N-21 dated: 28 October 2021).

## Guidelines and Standards Statement

This manuscript was prepared following the procedures outlined in Strengthening the Reporting of Observational Studies (STROBE) (Supplementary Materials S1). A complete list of reporting guidelines can be accessed via the Equator Network: https://www.equator-network.org/, accessed on 1 October 2025.

## References

[1] González D.S., Alvarado R.C. (2019). Envejecimiento de la Población y Cambio Climático.

[2] Casado J.M.R. (2023). Cambio climático, salud y persona mayor. An. RANM.

[3] Ayalon L., Keating N., Pillemer K., Rabheru K. (2021). Climate change and mental health of older persons: A human rights imperative. Am. J. Geriatr. Psychiatry.

[4] Fields L., Perkiss S., Dean B.A., Moroney T. (2021). Nursing and the sustainable development goals: A scoping review. J. Nurs. Scholarsh..

[5] Ortiz A.F. (2023). Salud planetaria. FMC Form..

[6] Álvarez-García C., López-Medina I.M., Sanz-Martos S., Álvarez-Nieto C. (2021). Salud planetaria: Educación para una atención sanitaria sostenible. Ed. Médica.

[7] Guzman C.A., Potter T., Aguirre A.A., Astle B., de Barros E.F., Bayles B.R., Chimbari M., El-Abbadi N., Evert J., Hackett F. (2021). The Planetary Health Education Framework.

[8] Lopez-Medina I., Álvarez-Nieto C., Grose J., Elsbernd A., Huss N., Huynen M., Richardson J. (2019). Competencies on environmental health and pedagogical approaches in the nursing curriculum: A systematic review of the literature. Nurse Ed. Pract..

[9] Portela dos Santos O., Perruchoud É., Pereira F., Alves P., Verloo H. (2024). Measuring nurses’ knowledge and awareness of climate change and climate-associated diseases: Systematic review of existing instruments. Nurs. Rep..

[10] American Association of Colleges of Nursing (2021). The Essentials: Core Competencies for Professional Nursing Education.

[11] White J., Gunn M., Chiarella M., Catton H., Stewart D. (2025). Renewing the Definitions of ‘Nursing’ and ‘A Nurse’. Final Project Report.

[12] Cugini F., Velez E.V., Gómez P.R. (2024). The climate crisis and human health: Survey on the knowledge of nursing students in the Comunidad de Madrid. Nurse Ed. Today.

[13] Atta M.H.R., Zoromba M.A., Asal M.G.R., Abdelhay E.S., Hendy A., Sayed M.A., Elmonem H.H.A., El-ayari O.S.M., Sehsah I., Abdelhay I.S. (2024). Predictors of climate change literacy in the era of global boiling: A cross-sectional survey of Egyptian nursing students. BMC Nurs..

[14] Abdelaziz F.S., Elzeiny A., Fouda N.M., Shahin M.A.H., Alabed H.H., Loutfy A. (2024). Nurses’ knowledge, skills, and attitudes regarding climate change and its impact on children’s health in Egyptian hospitals: A comparative study. Salud Cienc. Tecnol..

[15] Sibindi T., Chipps J.A., Crowley T. (2024). Eco-nursing competencies for nurses: A scoping review. Int. J. Nurs. Stud. Adv..

[16] Mani Z.A., Naylor K., Goniewicz K. (2025). Essential competencies of nurses for climate change response in Saudi Arabia: A rapid literature review. J. Adv. Nurs..

[17] Sayre L., Rhazi N., Carpenter H., Hughes N.L. (2010). Climate change and human health: The role of nurses in confronting the issue. Nurs. Adm. Q..

[18] Anderko L., Schenk E., Huffling K., Chalupka S. Climate Change, Health, and Nursing: A Call to Action. https://envirn.org/wp-content/uploads/2017/04/Climate-change-health-and-nursing-1-11-17.pdf.

[19] Middleton L., Howard A.A., Dohrn J., Von Zinkernagel D., Parham Hopson D., Aranda-Naranjo B., Hall C., Malata A., Bvumbwe T., Chabela A. (2014). The Nursing Education Partnership Initiative (NEPI) innovations in nursing and midwifery education. Acad. Med..

[20] McDermott-Levy R., Kolanowski A.M., Fick D.M., Mann M.E. (2019). Addressing the health risks of climate change in older adults. J. Gerontol. Nurs..

[21] Shaw E., Walpole S., McLean M., Alvarez-Nieto C., Barna S., Bazin K., Behrens G., Chase H., Duane B., Omrani O.E. (2021). AMEE Consensus Statement: Planetary health and education for sustainable healthcare. Med. Teach..

[22] Vandenbroucke J.P., von Elm E., Altman D.G., Gotzsche P.C., Mulrow C.D., Pocock S.J., Poole C., Schlesselman J.J., Egger M., STROBE Initiative (2014). The Strengthening the Reporting of Observational Studies in Epidemiology (STROBE) Statement: Guidelines for reporting observational studies. Int. J. Surg..

[23] Adam A.M. (2020). Sample size determination in survey research. J. Sci. Res. Rep..

[24] Lilienfeld E., Nicholas P.K., Breakey S., Corless I.B. (2018). Addressing climate change through a nursing lens within the framework of the united nations sustainable development goals. Nurs. Outlook.

[25] Álvarez-García C., Álvarez-Nieto C., Pancorbo-Hidalgo P.L., Sanz-Martos S., López-Medina I.M. (2018). Student nurses’ knowledge and skills of children’s environmental health: Instrument development and psychometric analysis using item response theory. Nurse Ed. Today..

[26] Álvarez-García C., Edra B., Marques G., Simões C., López-Franco M.D. (2025). Transcultural Adaptation of Environmental Health Questionnaire with Attitude, Knowledge, and Skills Scales for Portuguese Nursing Students. Nurs. Rep..

[27] Subiza-Pérez M., Vrotsou K., Esnal H., Kortajarena M., Mujika A., Marinelarena E., Aizpurua P., Arrue M., Mitxelena X., Larrinaga-Torrontegui U. (2024). Environmental health knowledge and competences in Basque health workers. A comparison of different professional profiles. Environ. Res..

[28] Iira T., Ruth M., Hannele T., Jouni J., Lauri K. (2021). Finnish nurses’ perceptions of the health impacts of climate change and their preparation to address those impacts. Nurs. Forum..

[29] Barimah A.J., Abdul-Ganiyu M., Dumba J., Commey R.D., Osei-Tutu A., Nketiah Y.B., Amoah B.O., Agyemang L., Kwadwo O., Yeboah G.O. (2025). Investigating health professionals’ understanding and risk perception of the effect of climate change on health. A cross-sectional study at three hospitals in Sunyani, Ghana. J. Public Health Res..

[30] Nsengiyumva R., Mukarubayiza M.R., Murekatete C., Meharry P. (2020). Climate change associated with neonatal health risks: Rwandan nurses and midwives’ awareness and perceptions. Rwanda J. Med. Health Sci..

[31] Vandenberg S.Y., Oosterbroek T., Chircop A., Kellett P. (2025). Registered Nurses’ Knowledge, Attitudes, and Practices Toward Climate-Sensitive Vector-Borne Diseases: Findings from a Cross-Sectional Survey. Public Health Nurs..

[32] Xiao J., Fan W., Deng Y., Li S., Yan P. (2016). Nurses’ knowledge and attitudes regarding potential impacts of climate change on public health in central of China. Int. J. Nurs. Sci..

[33] Yilma A.N., West K.P., Sauve H.E., Sznajder K.K., Hwang G., Baatiema L., Baatiema L., Kenu E., Noora C.L., Roba K.T. (2026). Frontline knowledge, attitudes, and practices on climate change and its link to zoonotic diseases: A mixed-methods study of healthcare workers in Ada East, Ghana. Discov. Public Health.

[34] Álvarez-García C., Álvarez-Nieto C., Sanz-Martos S., Puente-Fernández D., López-Leiva I., Gutiérrez-Puertas L., Canton-Habas V., Porcel-Galvez A.M., Lavedan-Santamaria A., Sarabia-Lavin R. (2019). Undergraduate nursing students’ attitudes, knowledge, and skills related to children’s environmental health. J. Nurs. Ed..

[35] Elsharkawy S.A., Elsheikh A.A., Refaat L.A.R. (2024). Knowledge, perception, and practices regarding climate change among students of Al-Azhar University for Girls in Cairo, Egypt. J. Public Health.

[36] Ali A.Z., Hassan A.K., Abd Elzaher O.M. (2024). Knowledge and Reported Practices of Nursing Students Regarding Health Effects of Climate Change in Sohag City. Assiut Sci. Nurs. J..

[37] Yang L., Liao W., Liu C., Zhang N., Zhong S., Huang C. (2018). Associations between Knowledge of the Causes and Perceived Impacts of Climate Change: A Cross-Sectional Survey of Medical, Public Health and Nursing Students in Universities in China. Int. J. Environ. Res. Public Health.

[38] Çetin A.Ö., Özdemir S.T. (2025). Climate Change Related Health Problems and Awareness of Nursing Students: A Cross-Sectional Study. Nurs. Health Sci..

[39] Aronsson J., Elf M., Warwick P., LoMartire R., Anåker A. (2025). The relevance of climate change and sustainability in nursing education: A cross-sectional study of students’ perspectives. BMC Nurs..

[40] Ryan E.C., Dubrow R., Sherman J.D. (2020). Medical, nursing, and physician assistant student knowledge and attitudes toward climate change, pollution, and resource conservation in health care. BMC Med. Ed..

[41] Kalogirou M.R., Dahlke S., Davidson S., Yamamoto S. (2020). Nurses’ perspectives on climate change, health and nursing practice. J. Clin. Nurs..

[42] Levine D.S., Strube M.J. (2012). Environmental attitudes, knowledge, intentions and behaviors among college students. J. Soc. Psychol..

[43] Byron L., Akerlof K.L. (2021). Climate and health concerns of Montana’s public and environmental health professionals: A cross-sectional study. BMC Public Health.

[44] Polivka B.J., Chaudry R.V., Mac Crawford J. (2011). Public health nurses’ knowledge and attitudes regarding climate change. Environ. Health Perspect..

[45] May K., Noel D. (2019). School nurses and climate change. Annu. Rev. Nurs. Res..

[46] Kallio H., Pietilä A., Kangasniemi M. (2020). Environmental responsibility in nursing in hospitals: A modified Delphi study of nurses’ views. J. Clin. Nurs..

[47] Álvarez-Nieto C., studen-García C., Parra-Anguita L., Sanz-Martos S., López-Medina I.M. (2022). Effectiveness of scenario-based learning and augmented reality for nursing students’ attitudes and awareness toward climate change and sustainability. BMC Nurs..

[48] Wilson R., Stanifer S.R., Wiggins A.T., Walton A.L. (2025). Oncology Nurses’ Awareness, Concern, Motivations, and Behaviors Related to Climate Change and Health. Clin. J. Oncol. Nurs..

[49] Anåker A., Nilsson M., Holmner Å., Elf M. (2015). Nurses’ perceptions of climate and environmental issues: A qualitative study. J. Adv. Nurs..

[50] Ozdemir C., Sahan S., Baykan N. (2026). The effect of environmental awareness on climate change worry and waste management in nurses: A cross-sectional study. J. Public Health.

[51] Kalogirou M.R., Dahlke S., Davidson S., Yamamoto S. (2021). How the hospital context influences nurses’ environmentally responsible practice: A focused ethnography. J. Adv. Nurs..

[52] Leffers J., Levy R.M., Nicholas P.K., Sweeney C.F. (2017). Mandate for the nursing profession to address climate change through nursing education. J. Nurs. Scholarsh..

[53] Beitz J., de Castro A.B. (2010). Integrating environmental health into nurse practitioner training-childhood pesticide exposure risk assessment, prevention, and management. AAOHN J..

[54] Kligler B., Pinto Zipp G., Rocchetti C., Secic M., Ihde E.S. (2021). The impact of integrating environmental health into medical school curricula: A survey-based study. BMC Med. Ed..

[55] Wong K.H., Allen A., Durrani T.S. (2019). Evaluating Effectiveness of Online Learning Modules in Pediatric Environmental Health Education. J. Med. Toxicol..

[56] Kihal Talantikite W., Renou D., Magdelaine A., Harang W., Zmirou-Navier D., Deguen S. (2020). Development of Serious Games (Equit’Game) to Address Health and Environmental Inequalities: Protocol for an App-Delivered Program to Perform a Territorial Diagnosis. JMIR Res. Protoc..

[57] İlaslan N., Orak N.Ş. (2025). Sustainable healthcare education using cooperative simulation in developing nursing students’ knowledge, attitude and skills: A randomized controlled study. Nurse Ed. Today.

